# Mesenchymal stromal cells as a therapeutic intervention for COVID-19: a living systematic review and meta-analysis protocol

**DOI:** 10.1186/s13643-021-01803-5

**Published:** 2021-09-15

**Authors:** Aidan M. Kirkham, Madeline Monaghan, Adrian J. M. Bailey, Risa Shorr, Manoj M. Lalu, Dean A. Fergusson, David S. Allan

**Affiliations:** 1 Department of Biochemistry, Microbiology & Immunology, University of Ottawa, Ottawa, Canada; 2 Clinical Epidemiology, Ottawa Hospital Research Institute, 501 Smyth Rd, Box 704, Ottawa, ON K1H 8L6 Canada; 3 School of Medicine, University of Ottawa, Ottawa, Canada; 4 Medical Information and Learning Services, The Ottawa Hospital, Ottawa, Canada; 5 Department of Cellular and Molecular Medicine, University of Ottawa, Ottawa, Canada; 6 Regenerative Medicine, Ottawa Hospital Research Institute, 501 Smyth Rdx, Box 704, Ottawa, ON K1H 8L6 Canada; 7 Department of Anesthesiology and Pain Medicine, The Ottawa Hospital, Ottawa, Canada; 8 School of Public Health and Epidemiology, Faculty of Medicine, University of Ottawa, Ottawa, Canada; 9 Department of Medicine, The Ottawa Hospital, Ottawa, Canada

**Keywords:** Mesenchymal stromal cells, Mesenchymal stem cells, MSCs, Extracellular vesicles, Exosomes, Microvesicles, Coronavirus disease 2019, COVID-19, Severe acute respiratory syndrome coronavirus 2, SARS-CoV-2, Acute respiratory distress syndrome, ARDS, Sepsis, Pneumonia

## Abstract

**Background:**

Mesenchymal stromal cells (MSCs) have significant immunomodulatory and tissue repair capabilities, mediated partly by conditioned media or through secreted extracellular vesicles (MSC-EVs). Infection with SARS-CoV-2 can cause mild to life-threatening illness due to activated immune responses that may be dampened by MSCs or their secretome. Many clinical studies of MSCs have been launched since the beginning of the global pandemic, however, few have been completed and most lack power to assess efficacy. Repeated systematic searches and meta-analyses are needed to understand, in real time, the extent of potential benefit in different patient populations as the evidence emerges.

**Methods:**

This living systematic review will be maintained to provide up-to-date information as the pandemic evolves. A systematic literature search of Embase, MEDLINE, and Cochrane Central Register of Controlled Trials databases will be performed. All clinical studies (e.g., randomized, pseudorandomized and non-randomized controlled trials, uncontrolled trials, and case series) employing MSCs or their secretome as a therapeutic intervention for COVID-19 will be included. Patients must have confirmed SARS-CoV-2 infection. Study screening and data extraction will be performed in duplicate. Information concerning interventions, patient populations, methods of MSC isolation and characterization, primary and secondary clinical and/or laboratory outcomes, and adverse events will be extracted. Key clinical outcomes will be pooled through random-effects meta-analysis to determine the efficacy of MSCs and their secreted products for COVID-19.

**Discussion:**

Our systematic review and subsequent updates will inform the scientific, medical, and health policy communities as the pandemic evolves to guide decisions on the appropriate use of MSC-related products to treat COVID-19.

**Systematic review registration:**

PROSPERO CRD 42021225431

**Supplementary Information:**

The online version contains supplementary material available at 10.1186/s13643-021-01803-5.

## Introduction

Severe acute respiratory syndrome coronavirus 2 (SARS-CoV-2), which is responsible for coronavirus disease 2019 (COVID-19), is a pathogenic beta coronavirus that has now spread to over 200 countries around the world and infected millions of people [1]. Cells and tissues infected with SARS-CoV-2 experience a rapid recruitment of pro-inflammatory immune cells to the affected tissue [2]. The ensuing surge of inflammatory cytokine production (termed “cytokine storm”), causes significant tissue damage through apoptosis and pyroptosis of cellular constituents [3, 4]. A large portion of patients who contract COVID-19 remain asymptomatic or experience mild symptoms [5]. However, many patients experience more severe symptoms [6]. These severe symptoms can manifest as serious respiratory complications such as acute lung injury [7], pneumonia [8], pulmonary fibrosis [9] and acute respiratory distress syndrome (ARDS) [10], or extrapulmonary complications including thrombosis [11], myocarditis [12], pericarditis [13], liver dysfunction [14], acute kidney injury [15], sepsis [16], and multiorgan failure [17].

Since its rapid emergence in December 2019, researchers from across the globe have been working to develop strategies to combat COVID-19. In an attempt to discover effective COVID-19 therapeutics, several living systematic reviews and network meta-analyses have been performed on a variety of repurposed therapeutic agents with diverse mechanisms of action. Some agents including hydroxychloroquine, lopinavir–ritonavir, and convalescent plasma have demonstrated little to no benefit over standard of care in terms of improving important clinical outcomes such as all-cause mortality, duration of hospitalization, and risk of requiring mechanical ventilation [18, 19]. Other therapeutic agents including dexamethasone, glucocorticoids, remdesivir, and tocilizumab have demonstrated benefits over standard of care in terms of improving important clinical outcomes such as all-cause mortality, risk of requiring mechanical ventilation, and progression of clinical symptoms [18–20]. However, the magnitude of these benefits is marginal at best, and the quality of most studies included in these meta-analyses was generally low, drastically reducing the certainty in these observed modest effects [18–20]. Thus, a proven safe and effective therapeutic agent for combatting COVID-19 has yet to be discovered.

One potentially useful therapy for COVID-19 is mesenchymal stromal cells (MSCs) [21–26]. MSCs are multipotent stem cells that can be isolated from a variety of adult and neonatal tissues [27]. Several preclinical and clinical studies have demonstrated that MSCs possess remarkable immunomodulatory properties and the capacity for tissue repair [28–35]. Additionally, MSCs are poorly immunogenic and elicit minimal immune response when administered to patients [36, 37], allowing for third party “off the shelf” use. Furthermore, the occurrence of serious adverse events associated with MSC therapy is rare [38–40]. MSCs may achieve their immunomodulatory and regenerative capabilities without cell engraftment through paracrine mechanisms [41], including MSC-derived extracellular vesicles (MSC-EVs) [42] and conditioned medium (MSC-CM) [43].

The rapid pace of the evolving COVID-19 pandemic combined with ongoing issues such as variants of concern and the potential for illness in vaccinated individuals means that systematic reviews can become outdated quickly, underscoring the need for a living systematic review that can be updated regularly [44]. The use of the living systematic review framework will provide researchers, clinicians, and policymakers with the most up-to-date information surrounding the use, efficacy, and safety of MSCs and their secretome as therapeutic interventions for COVID-19 [44].

### Research objectives

This review will examine all clinical studies of MSCs or their secretome (MSC-EVs, MSC-CM) as a therapeutic intervention for COVID-19. Our primary objective is to assess the improvement of clinical symptoms and outcomes in patients with confirmed SARS-CoV-2 infection who have been administered MSCs or their secretome as a therapeutic intervention for COVID-19. Our secondary objective is to describe how study designs and aspects of MSC manufacturing and administration differ across the studies and the extent to which these differences impact patient outcomes (e.g., MSC tissue source, dosage, route of administration, patient characteristics, severity of COVID-19, co-morbidities), and to describe adverse events associated with MSC treatment. Given the expected heterogeneity of methods used (e.g., cell sources, isolation techniques) and variability in outcomes assessed across studies, a systematic review and meta-analysis is needed to provide context on the effectiveness of this therapy and how MSC treatment can be refined to optimize patient outcomes.

## Methods and design

### Protocol

This systematic review protocol is reported in accordance with the Preferred Reporting Items for Systematic reviews and Meta-Analysis Protocol (PRIMSA-P) reporting guidelines [45] (see Additional file 1). A summary of the protocol has been registered at the International Prospective Registry of Systematic Reviews (PROSPERO CRD 42021225431). The first systematic search of the literature is planned for February 3rd, 2021. An updated search will be performed 6 months after this initial search (August 3rd, 2021) to capture any new articles that have been published. A third and final update to the search will be performed on February 3rd, 2022 (6 months after the second search, 12 months after the first search).

## Eligibility criteria

### Population

Inclusion — Patients of any age (adults or pediatrics) with suspected and/or confirmed SARS-CoV-2 infection (by nucleic acid amplification testing, antibody assay, or antigen testing) who are experiencing symptoms of coronavirus disease (COVID-19) or asymptomatic patients with confirmed SARS-CoV-2 infection.

Exclusion — Studies of patients without confirmed SARS-CoV-2 infection will be excluded.

### Intervention

Inclusion — We will include studies of MSCs or their secretome to treat COVID-19 in symptomatic or asymptomatic patients with confirmed SARS-CoV-2 infection. MSCs or their secretome can be derived from any tissue source (e.g., bone marrow, adipose, umbilical cord, dental pulp, placenta, etc.). Tissues from which MSC/secretome are obtained may be syngeneic, allogeneic, or xenogeneic. All routes of MSC/secretome administration will be considered (intravenous injection, aerosol inhalation, intramuscular injection, etc.). MSCs/secretome may be administered along with other therapeutic agents (antivirals, anti-cytokine drugs, immunomodulatory agents, etc.).

Exclusion — Studies in which only non-MSC-based therapeutics are administered to treat COVID-19, such as non-MSC cells (natural killer cells, T cells, artificial antigen-presenting cells), antivirals (remdesivir, sofosbuvir, ribavirin), immunomodulatory drugs (dexamethasone, sarilumab, baricitinib), combination therapies that do not include MSC-based treatment, and anti-cytokine drugs (anti-IL-6, anti-IL-1, anti-TNF-α, etc.). Studies that administered concomitant treatments along with MSC-based therapies will be included.

### Comparators

Patients receiving conventional therapies for COVID-19 treatment (antivirals, immunomodulatory drugs, anti-cytokine drugs, combination therapies, etc.) or placebo will be included. Studies that do not include a comparator arm (i.e., Single-arm studies, observational studies) will also be included and may be analyzed or described separately from studies with comparator arms, as appropriate.

### Outcomes

The primary outcomes for this study are overall mortality, the need for ICU admission, and the need for mechanical ventilation. These outcomes will be measured post-intervention (i.e., after administration of MSCs, MSC secretome, or comparator treatments). Dichotomous outcome measures will be expressed as risk ratios at the study endpoint (RR).

The secondary outcomes for this study include COVID-19 severity according to the World Health Organization Ordinal Scale for Clinical Improvement (WHO-OSCI) [46], length of time in the intensive care unit (ICU), length of time on mechanical ventilation, length of time in hospital, presence and severity of clinical symptoms (fever, cough, shortness of breath, chest pain, etc.), presence and size of pulmonary lesions (CT scan) and change in oxygenation levels (e.g., PaO2/FiO2 ratio), viral load, body temperature, organ failure assessment score (e.g., SOFA), circulating levels of immune cells (lymphocytes, neutrophils, macrophages, regulatory dendritic cells, NK cells), pro-inflammatory cytokines, (IL-6, TNF-α, IFN-γ, etc.), anti-inflammatory cytokines (IL-10, TGF-β, etc.) and inflammatory markers (C-reactive protein, Ferritin, D-dimer, etc.), and adverse events arising from MSC/secretome administration (tumorigenesis, thromboembolism, ectopic tissue formation, etc.). These outcomes will be measured pre-intervention and post-intervention (intervention = administration of MSCs or MSC secretome). Dichotomous, endpoints will be expressed as risk ratios (RR) and continuous endpoints will be expressed as mean difference (MD) or standardized mean difference (SMD).

### Study types

Inclusion — All clinical studies (controlled and uncontrolled) examining the use of MSCs or their secretome as a therapeutic intervention for COVID-19 will be included. Studies may be randomized, pseudo-randomized, or non-randomized. Studies may be single-armed, observational, or have a comparator or control.

### Data sources

We will search Embase Classic+Embase, Ovid MEDLINE(R), Ovid EBM Reviews, and Cochrane Central Register of Controlled Trials from inception onwards (Additional file 2). Preprint servers (e.g., medRxiv) will not be searched for this review. Other sources that become available during future iterations of the search will be considered, including search platforms that are specific for COVID-19. Additionally, two reviewers (AMK, MM) will perform manual searches of the bibliographies of included articles and relevant reviews will be performed.

### Search strategy

The search strategies (Additional file 2) used for this review will be generated by a health sciences librarian (RS) with experience designing systematic literature searches. A second information specialist with no association to the project will also review the strategy using Peer Review of Electronic Search Strategies (PRESS) before the final search procedure is executed [47, 48]. The search strategy will utilize calculated vocabulary and MeSH terms (e.g., mesenchymal stem cells, mesenchymal stromal cells, mesenchymal stromal cell extracellular vesicles, mesenchymal stromal cell conditioned medium, severe acute respiratory syndrome coronavirus 2, coronavirus disease 2019, acute respiratory distress syndrome) and acronyms (e.g., MSCs, MSC-EVs, MSC-CM, SARS-CoV-2, COVID-19, ARDS), with alterations as required per database. No language or publication date restrictions will be applied for the initial search. Keywords for the search will include mesenchymal stromal cells, mesenchymal stem cells, MSCs, mesenchymal stromal cell conditioned medium, MSC-CM, mesenchymal stromal cell extracellular vesicles, MSC-EVs, exosomes, microvesicles, coronavirus disease 2019, COVID-19, severe acute respiratory syndrome coronavirus 2, SARS-CoV-2, acute respiratory distress syndrome, ARDS, sepsis, pneumonia. The search will be updated every 6 months to capture any new relevant citations for inclusion in future iterations of this living systematic review. Unpublished studies, abstracts, review articles, editorials, and letters will be excluded.

## Study records

### Data management

All citations obtained from the systematic search of the literature will be uploaded to Rayyan QCRI systematic review software (https://www.rayyan.ai/) [49].

### Selection process

Two reviewers (AMK, MM) will screen the titles and abstracts in an independent and blinded fashion using the a priori inclusion and exclusion criteria outlined above. Reasons for study exclusion during this initial screening will not be recorded. Studies that appear to meet the inclusion criteria, or where there is any uncertainty, the full-text articles will be obtained. The same two reviewers (AMK, MM) will assess the eligibility of full-text articles in an independent and blinded fashion using the same a priori inclusion and exclusion criteria. Any disagreement between the two reviewers will be resolved through discussion with a third-party member (DSA). Reasons for study exclusion at this level will be recorded. Study screening for updates to this review will shortly after the search is completed. For our initial iteration of the review, all clinical studies (both controlled and uncontrolled), examining the use of MSCs or their secretome as a therapeutic intervention for COVID-19 will be included. As more studies become available, future iterations of the review will utilize only controlled studies, with the eventual goal being to use only randomized controlled trials (RCTs).

### Data collection process

A standardized data extraction form will be designed in Microsoft Excel (Microsoft Corporation, Seattle, USA) by the primary review author (AMK). Relevant information from each study will be extracted from each eligible study by two reviewers (AMK, MM) in an independent and blinded fashion. After data extraction is complete, any disagreements will first be discussed between the two independent reviewers (AMK, MM). If a resolution cannot be reached, a third-party member will be consulted (DSA).

### Data items

Broad categories of data items to be extracted include study characteristics, baseline patient characteristics (age and gender breakdown, severity of COVID-19, co-morbidities, etc.), intervention details (MSC tissue source, dose, route of administration, timing of administration, etc.), methods of MSC/secretome isolation and characterization (ultracentrifugation, scanning electron microscopy, western blot, flow cytometry, etc.), whether MSCs met the ISCT criteria [50] (plastic adherence, trilineage differentiation potential, positive and negative surface markers), primary and secondary clinical outcomes, and adverse events arising from MSC therapy.

### Risk of bias assessment

The Cochrane risk of bias tool (RoB) for randomized trials version (2.0) will be used to assess the risk of bias for all included randomized studies (RCTs) [51]. The ROBINS-I tool for non-randomized studies will be used to assess the risk of bias for all other interventional controlled studies [52]. The EBM tool will be used to assess study quality in observational or uncontrolled studies [53]. Risk of bias assessment will be assessed at the study level by two independent, blinded reviewers (AMK, AJMB). Any discrepancies will be resolved by a third reviewer (DSA).

### Confidence in cumulative evidence

We will use the GRADE criteria [54] to rate the strength of the body of evidence with regards to the effect of MSCs and their secretome on our primary outcome (mortality). This will strengthen the quantitative and narrative conclusions of the review, which will allow scientists, clinicians, and policymakers to make informed decisions regarding the design of future clinical studies, regulatory approval, and clinical translation of this novel cellular therapy.

### Data analysis

Meta-analysis is planned should there be enough controlled studies reporting our primary outcomes. Data will be pooled from multiple studies if there are three or more studies that report the same outcome. Data synthesis and analysis will be performed using RevMan 5 (Version 5.4) Systematic Review Software (https://training.cochrane.org/online-learning/core-software-cochrane-reviews/revman/revman-5-download). All primary outcomes will be analyzed and plotted separately as forest plots. Outcomes that will be attempted to be synthesized will include mortality rate, number of patients requiring ICU admission, and number of patients requiring mechanical ventilation. Because all primary outcomes are dichotomous variables (mortality rate, number of patients requiring ICU admission, number of patients requiring mechanical ventilation), the results from each study will be pooled and described as risk ratios (RR) with 95% confidence intervals using the DerSimonian and Laird random-effects method [55]. Before a meta-analysis is executed, the number of included studies and the outcomes they report will be assessed in consultation with a senior team member (DSA, DAF). If a sufficient number of studies exist with homogeneity in terms of outcome reporting, a meta-analysis will be performed. Statistical heterogeneity of effect sizes will be assessed using the I^2^ statistic [56]. The thresholds for interpretation of are *I*^2^: 0–40% (low heterogeneity), 30–60% (moderate heterogeneity), 50–90% (substantial heterogeneity), and 75–100% (considerable heterogeneity) [56]. Should considerable heterogeneity exist, sources of heterogeneity will be explored through subgroup analysis. Evidence tables will be used to provide an overall description of included studies with regards to characteristics such as study design, participants, and intervention/control groups [57]. Furthermore, evidence tables will be used to provide an overview of the number of studies reporting our primary/secondary outcomes and the number of studies reporting the occurrence of adverse events [57]. New data from subsequent searches of the literature will be incorporated into the review upon publication. The review authors will manually generate figures and update the results section of the review accordingly.

### Subgroup analyses

Subgroup analysis is planned should a sufficient number of studies exist. Planned product-related subgroup analyses include efficacy of MSCs isolated from different tissue sources (bone marrow, adipose, umbilical cord blood, dental pulp, etc.), MSCs compared to their secretome (MSC-EVs, MSC-CM), and MSCs that have been treated (chemical agents, hypoxia, etc.) and/or genetic modified (e.g., altered expression of specific RNA species) before administration or isolation of paracrine factors. Planned patient-related subgroup analyses will address the efficacy of MSCs or their secretome according to COVID-19 severity (moderate, severe, critical), presence of co-morbidities (no-comorbidities, one co-morbidity, multiple co-morbidities), patient age (over 55 vs under 55), and patient sex (male vs female). In addition, we will plan to analyze subgroups based on geographic location (China, Europe, North America, other) and by type of funding support for the study (public funding via government or charity, private funding from industrial sponsor, other).

### Meta-biases assessment

Evaluation for publication bias will be conducted using a funnel plot [58]. These funnel plots will be generated using RevMan 5 (Version 5.4) Systematic Review Software (https://training.cochrane.org/online-learning/core-software-cochrane-reviews/revman/revman-5-download).

### Knowledge dissemination

Our findings will be disseminated through a widely read, open access, peer-reviewed scientific journal (e.g., PLOS Medicine, JAMA). Furthermore, we will share our findings at scientific meetings (e.g., American Society for Transplantation and Cellular Therapy (ASTCT) meetings, Till & McCulloch meetings (Stem Cell Network)) and through the preparation of evidence briefs that will be shared with health policy groups including Health Canada and the World Health Organization.

### Living systematic review approach

Living systematic reviews are defined by Cochrane as a method of updating systematic reviews frequently to incorporate new evidence regarding an intervention of interest as soon as it becomes available [59]. While living systematic reviews and non-living systematic reviews both adhere to standard rigorous systematic review methodology (e.g., screening, data extraction, and risk of bias assessment), living systematic reviews also include explicit, transparent, and pre-specified decisions on how frequently the literature will be searched for new evidence and when new updates of the review are expected to be published [59]. Several criteria should be met to ensure that the intervention under consideration is well suited for performing a living systematic review [59]. For a living systematic review to be performed, the review question should be a priority for decision-making, there should be considerable uncertainty surrounding the existing evidence, and there should be a substantial chance that emerging evidence will impact the conclusions of the review [59]. In the case of MSCs and their secretome as a therapeutic intervention for COVID-19, these pre-specified criteria would be met, making this topic well suited for a living systematic review. There remains an urgent need for clarity regarding optimal management and many ongoing studies are actively recruiting and are anticipated to contribute to the evolving evidence base over the next 12 months. Additionally, the skill level and workload for the review team should be considered carefully before initiating the review to ensure that the appropriate personnel are available to support the ongoing maintenance of the living systematic review [59]. The bulk of project management will be undertaken by a graduate student (AMK) who is completing the review as the main component of their graduate studies. Our review team also has access to several medical students who will be available to perform tasks which must be done in duplicate (MM, AJMB) for the duration of the review. Furthermore, a medical information specialist (RS) is also available for the whole expected duration of the project. Lastly, our team has several experienced senior members (DSA, DAF, MML) who can assist in moving the review forward and offering advice should problems arise.

### Updates

**Fig. 1:**
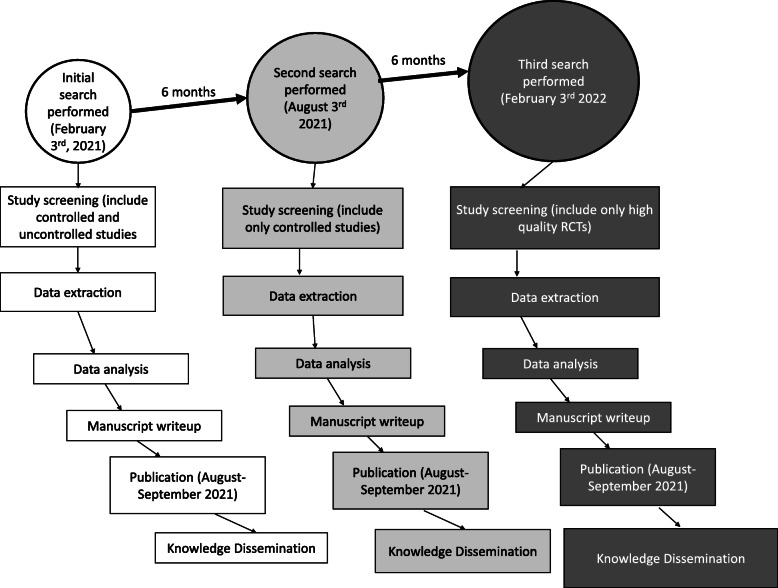
Diagram of anticipated updates of the living systematic review. Updates will increase the quantity and quality of evidence to refine conclusions

The literature search for this living review will be updated every 6 months (see Fig. 1). Article screening and data analysis will be performed immediately following updates to the literature search. The review itself will be updated every 6 months or as required depending on the amount of new evidence that emerges. New data will be incorporated into the review upon publication. The review authors will manually generate all figures/tables and update the results section of the review accordingly. We will not utilize any techniques to update this living systematic review in a rapid manner (e.g., machine learning) as soon as individual studies are published.

### Amendments

If any amendments to this protocol are necessary, the date and specific changes to the protocol will be documented on PROSPERO (CRD42021225431) with rationales as to why the alterations were required.

## Discussion

This systematic review will summarize all published clinical studies investigating the safety and efficacy of MSCs and their secretome as a therapeutic intervention for COVID-19. Due to their immunomodulatory and regenerative capabilities, MSCs and their secretome have the potential to dampen the inflammatory immune response initiated by SARS-CoV-2 infection and heal damaged tissues [60, 61]. MSC-EVs and MSC-CM represent a particularly promising approach to harness the therapeutic benefits of MSCs with lower manufacturing/storage costs and an improved safety profile [41–43]. Of particular significance, MSCs and their secretome have demonstrated promise for a number of COVID-19-related ailments including acute respiratory distress syndrome (ARDS) [62], acute lung injury [63], pneumonia [64], pulmonary fibrosis [65], acute kidney injury [66], myocarditis [67], and sepsis [68].

Despite extensive funding allocation and research, only two therapeutics are approved to date, for the treatment of COVID-19 in Canada [69], Bamlanivimab, a monoclonal antibody directed against the spike protein of SARS-CoV-2 [70], and Remdesivir, a broad-spectrum adenosine analog [71]. Additionally, three antibody cocktails have been granted emergency use authorization in the USA: bamlanivimab and remdesivir, bamlanivimab combined with etesevimab, and REGEN-COV (casirivimab and imdevimab) [72]. Although remdesavir effectively reduces the recovery time of COVID-19 patients [73], it is only approved for use in severe COVID-19 patients [71]. In contrast, although bamlanivimab and other antibody therapies prevent the worsening of COVID-19 symptoms and reduce hospitalizations [74], their use is currently restricted to patients with mild–moderate COVID-19 that are at high risk of progressing to severe COVID-19 [70]. Furthermore, both these therapies are not approved for use in pediatric populations [70, 71], are resource intensive [75, 76], and may be susceptible to escape mutants [77, 78].

Though living systematic reviews are important for disseminating critical information in a timely fashion, they require a significant commitment from the primary author and the rest of the review team to keep up to date with rapidly evolving bodies of literature [59]. This is particularly true for COVID-19 related reviews as the rate at which new studies are being published [79] may make it challenging to keep pace with the evolving knowledge base and provide readers with versions of the review which incorporate all relevant evidence at any given timepoint. Though our review team currently possesses the required resources to maintain a living systematic review of this nature, circumstances could change that affect our ability to update the review in the prespecified time intervals laid out in this protocol. Furthermore, with the uncertainty surrounding the timeline of the COVID-19 pandemic, it may be necessary to transition this review from a living systematic review to a standard systematic review should the rate at which studies in this area are being published fall dramatically. At this point, we will follow the guidelines recommended by Cochrane for transitioning out of a living systematic review for our final update of the review to inform readers that the review is no longer in living mode [59].

Our planned systematic review and meta-analysis is not without limitations. Firstly, informative practice-changing systematic reviews and meta-analyses often incorporate many high-quality studies with an overall patient population large enough to achieve the statistical power necessary to detect differences between control and experimental groups [80, 81]. Although a large number of trials examining the use of MSCs and/or their secretome as a therapeutic intervention for COVID-19 have been registered across clinical trial databases globally [82], most of the published literature to this point is in the form of case reports, case series, uncontrolled studies, or small early-phase controlled trials. With these low-quality trials appearing to make up the bulk of published studies at this point, it is possible that this review will not produce any meaningful conclusions, especially the first iteration. Secondly, the rapid onset of the pandemic has led to a rush by researchers to register, run and publish trials without following robust clinical trial methodology [83]. This lack of methodological rigor may increase the risk of bias of the individual studies included in this review, which will in turn decrease the certainty of the conclusions of the review as a whole [84]. Lastly, with the duration of the pandemic being unknown and vaccine rollout well underway in many countries, it is possible that all these registered trials may never enroll enough patients to reach completion. Thus, the possibility exists that there may never be enough high-quality controlled studies published to determine the true extent to which MSCs and their secretome are an effective therapeutic intervention for COVID-19.

To diversify the available therapeutic options and fill unmet therapeutic needs, there has been an enormous surge in the number of registered clinical trials examining COVID-19-related interventions. Among these registered trials, several studies examining the use of MSC-based therapies for COVID-19 can be found. However, significant heterogeneity can be observed in terms of the study design, intervention characteristics, and outcome reporting across these trials. This systematic review will capture this heterogeneity and discern the most frequently employed and effective approaches. A meta-analysis of the primary outcome will allow for a better interpretation of the true clinical efficacy of MSCs and their secretome as a therapeutic intervention for COVID-19 than could be achieved in any individual study alone. With the majority of studies not planning to enroll enough patients to reach adequate statistical power to determine efficacy, the findings presented in this review should be useful in guiding the design and implementation of more definitive clinical trials in the future or may possibly provide sufficient evidence to support approval of this class of cell-based therapy. Taken together, our planned systematic review and meta-analysis will help accelerate approval of this novel cellular therapy by relevant regulatory bodies and propel MSC-based therapy for COVID-19 towards use in the broader population should there be sufficient evidence demonstrating its safety and efficacy.

## Supplementary Information

**Additional file 1.** PRISMA-P Checklist.

**Additional file 2.** Search Strategy.

## Data Availability

Not applicable at this stage. Datasets generated from the proposed work will be available upon request from the corresponding author.
