# Supplementary material for: Mesenchymal stromal cells as a therapeutic intervention for COVID-19: a living systematic review and meta-analysis protocol
Source: Syst Rev. 2021 Sep 15;10:249. doi: 10.1186/s13643-021-01803-5 (PMC8441251; doi:10.1186/s13643-021-01803-5)
Supplement: Supplementary file 2 — Additional file 2. Search Strategy. [file 13643_2021_1803_MOESM2_ESM.docx]

Database: Embase Classic+Embase <1947 to 2021 February 03>, Ovid MEDLINE(R) ALL <1946 to February 03, 2021>, EBM Reviews - Cochrane Central Register of Controlled Trials <December 2020>

Search Strategy:

--------------------------------------------------------------------------------

1 COVID-19/ (17886)

2 (exp coronavirus/ or coronavirus*.mp. or corona virus*.mp.) and (wuhan or beijing or shanghai).mp. (9261)

3 ((coronavirus or corona virus) adj3 "2019").tw. (41532)

4 (covid or covid2019).tw,kf. (175983)

5 covid19.tw,kw. or covid 19.kf. (42778)

6 sars cov 2.tw,kw. (62711)

7 (ncov or n cov).tw,kw. (3072)

8 novel coronavirus.tw,kw. (14551)

9 sars cov2.tw,kw. (3114)

10 Coronavirus Infections/ and Pandemics/ (39146)

11 (ncov19 or ncov-19 or 2019-novel CoV).tw,kf. (119)

12 or/1-11 (198342)

13 Mesenchymal Stromal Cells/ (49672)

14 Mesenchymal Stem Cell Transplantation/ (24831)

15 Multipotent Stem Cells/ (8889)

16 (MSC or MSCs or ADMSC or ADMSCs or BM-MSC or BM-MSCs or BMD-MSC or BMD-MSCs or BMDMSC or BMDMSCs).tw,kw. (88902)

17 (mesenchymal adj5 (cell* or stem or stromal or progenitor* or multipotent or bone marrow or adipose or placenta*)).tw. (199459)

18 (mesenchymal and (cell* or stem or stromal or progenitor or multipotent or bone marrow or adipose or placenta*)).kw. (33543)

19 ((multipotent or multi-potent) adj (stroma* cell* or stem cell*)).tw,kf. (4692)

20 (msc ev or msc cm).tw,kf. (1071)

21 (colony-forming adj2 fibroblast*).tw. (1675)

22 marrow stroma* cell*.tw. (17531)

23 Mesoderm/cy (5853)

24 Exosomes/ or Extracellular Vesicles/ (42505)

25 exosom*.tw,kw. (41425)

26 Cell-Derived Microparticles/ (8876)

27 (microvesicle* or micro vesicle*).tw,kw. (11432)

28 ((cell or cells or extracellular) adj2 vesicle*).tw. (29239)

29 microparticle*.tw,kw. (42112)

30 (extracellular and vesicle*).kf. (5278)

31 ((cell or cells) and vesicle*).kf. (2195)

32 or/13-31 (354252)

33 12 and 32 (743)

**34 33 use medall (306) Medline**

35 (Coronavirinae/ or coronavirus*.mp. or corona virus*.mp.) and (wuhan or beijing or shanghai or hubei).mp. (9894)

36 ((coronavirus* or corona virus* or coronavirus* or coronaviridae or coronaviridae or betacoronavirus*) adj3 ("19" or "2019")).tw. (52354)

37 (covid or covid19).tw. (173537)

38 sars cov 2.tw. (53939)

39 (ncov or n cov).tw. (2696)

40 (novel coronavirus* or novel corona virus*).tw. (14310)

41 (CoV 2 or CoV2 or sarscov2 or 2019nCoV or novel CoV or wuhan virus).tw. (56040)

42 (coronavirus infection/ or severe acute respiratory syndrome/) and (pandemic/ or pandemic*.tw.) (51355)

43 limit 42 to yr="2019 -Current" (50254)

44 35 or 36 or 37 or 38 or 39 or 40 or 41 or 43 (197531)

45 exp mesenchymal stem cell/ (102855)

46 exp mesenchymal stem cell transplantation/ (24831)

47 exp mesenchymal stroma cell/ (14237)

48 (MSC or MSCs or ADMSC or ADMSCs or BM-MSC or BM-MSCs or BMD-MSC or BMD-MSCs or BMDMSC or BMDMSCs or BMSC or BMSCs or HBMSC or HBMSCs or ABMSC or ABMSCs or MAPC or MAPCs).tw. (106309)

49 (((multipotent or multi-potent) adj3 (stem or stroma$1 or progenitor*)) and (cell$1 or cell-induced)).tw. (16261)

50 multipotent stem cell/ (9199)

51 (mesenchymal adj5 (cell* or stem or stroma* or progenitor* or multipotent or bone marrow or adipose or placenta*)).tw. (199944)

52 (colony-forming adj2 fibroblast*).tw. (1675)

53 (marrow stroma* adj2 cell*).tw. (18363)

54 exosome/ (31880)

55 exosom*.tw. (37895)

56 (microvesicle* or micro vesicle*).tw. (10182)

57 microparticle*.tw. (40494)

58 ((cell or cells or extracellular) adj2 vesicle*).tw. (29239)

59 exp membrane microparticle/ (8167)

60 (msc ev or msc cm).tw. (1070)

61 or/45-60 (359808)

62 44 and 61 (735)

**63 62 use emczd (370) Embase**

64 COVID-19/ (17886)

65 (exp coronavirus/ or coronavirus*.mp. or corona virus*.mp.) and (wuhan or beijing or shanghai).mp. (9261)

66 ((coronavirus or corona virus) adj3 "2019").tw. (41532)

67 (covid or covid2019).tw,kw. (175722)

68 covid19.tw,kw. or covid 19.kw. (75538)

69 sars cov 2.tw,kw. (62711)

70 (ncov or n cov).tw,kw. (3072)

71 novel coronavirus.tw,kw. (14551)

72 sars cov2.tw,kw. (3114)

73 Coronavirus Infections/ and Pandemics/ (39146)

74 (ncov19 or ncov-19 or 2019-novel CoV).tw,kw. (125)

75 or/64-74 (199082)

76 Mesenchymal Stromal Cells/ (49672)

77 Mesenchymal Stem Cell Transplantation/ (24831)

78 Multipotent Stem Cells/ (8889)

79 (MSC or MSCs or ADMSC or ADMSCs or BM-MSC or BM-MSCs or BMD-MSC or BMD-MSCs or BMDMSC or BMDMSCs).tw,kw. (88902)

80 (mesenchymal adj5 (cell* or stem or stromal or progenitor* or multipotent or bone marrow or adipose or placenta*)).tw. (199459)

81 (mesenchymal and (cell* or stem or stromal or progenitor or multipotent or bone marrow or adipose or placenta*)).kw. (33543)

82 ((multipotent or multi-potent) adj (stroma* cell* or stem cell*)).tw,kw. (4749)

83 (msc ev or msc cm).tw,kw. (1070)

84 (colony-forming adj2 fibroblast*).tw. (1675)

85 marrow stroma* cell*.tw. (17531)

86 Mesoderm/cy (5853)

87 Exosomes/ or Extracellular Vesicles/ (42505)

88 exosom*.tw,kw. (41425)

89 Cell-Derived Microparticles/ (8876)

90 (microvesicle* or micro vesicle*).tw,kw. (11432)

91 ((cell or cells or extracellular) adj2 vesicle*).tw. (29239)

92 microparticle*.tw,kw. (42112)

93 (extracellular and vesicle*).kw. (5106)

94 ((cell or cells) and vesicle*).kw. (2780)

95 or/76-94 (354775)

96 75 and 95 (739)

**97 96 use cctr (79) Cochrane**

98 34 or 63 or 97 (755)

99 remove duplicates from 98 (487)

100 99 use medall (295)

101 99 use emczd (115)

102 99 use cctr (77)
